# Supplementary material for: Emotional Exhaustion, a Proxy for Burnout, Is Associated with Sleep Health in French Healthcare Workers without Anxiety or Depressive Symptoms: A Cross-Sectional Study
Source: J Clin Med. 2023 Feb 27;12(5):1895. doi: 10.3390/jcm12051895 (PMC10004252; doi:10.3390/jcm12051895)
Supplement: Supplementary file 1 [file jcm-12-01895-s001.zip › jcm-2146142-supplementary.pdf]

Supplementary Files

Figure S1. Adjusted linear associations between emotional exhaustion and sleep health.

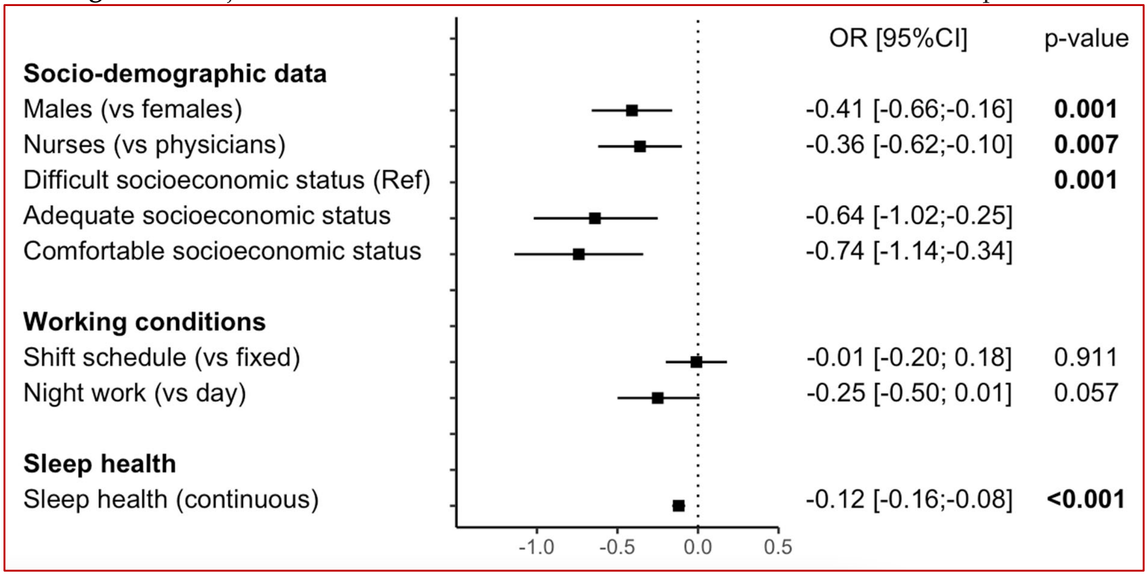

Forest plot of the associations with emotional exhaustion (continuous) after adjustment for age, sex, job category, perceived socioeconomic status, work schedule, night work, telecommuting, work in a COVID-19 unit, self-perceived health and sleep health.

Figure S2. Adjusted associations between emotional exhaustion and sleep health with additional adjustment for anxiety and depressive symptoms.

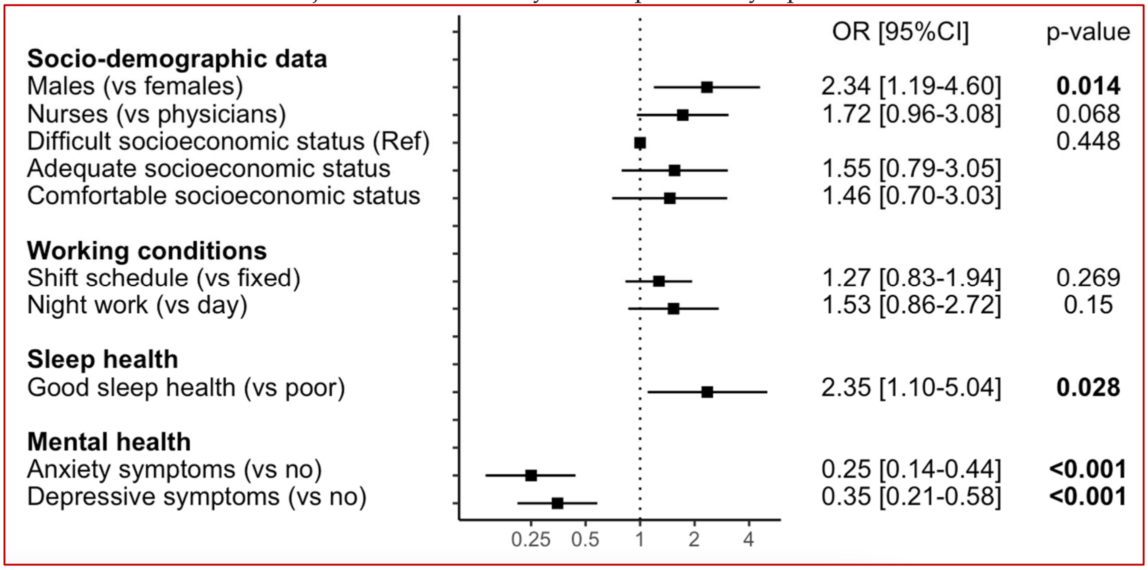

Forest plot of the associations with the absence of emotional exhaustion after adjustment for age, sex, job category, perceived socioeconomic status, work schedule, night work, telecommuting, work in a COVID-19 unit, self-perceived health, sleep health and anxiety and depressive symptoms and their interaction term.
